# Class-Room Talks, No. 2

**Published:** 1888-01

**Authors:** J. T. Kent

**Affiliations:** St. Louis


### CLASS-ROOM TALKS. No. 2.

Professor J. T. Kent, M. D., St. Louis.

Headaches are quite manageable, and students going out from *this* college ought to be quite able to manage all chronic cases in the neighborhood where they settle. The greatest trouble will be found in persuading the patients to co-operate with you. These patients will come to you for *relief*. This headache has been a long standing trouble to themselves. And many have been in this family for a generation or two. They do not expect to be cured, and have been accustomed to treating themselves with Morphine, Chloroform, Chloral, or one of the Bromides, which has palliated or suppressed the pain for the time being. And they will expect you to do the same thing for them. It will be hard to persuade them to go home, *stop all medication* and *let the symptoms develop*. An intelligent person will do so *if* they have the matter carefully explained to them, showing them that it is impossible to apply the *law of similars* unless you *know the symptoms* for which you are expected to prescribe. Sometimes it will be necessary to resort to a period of Sac. Lac., but it is much better to tell them plainly, where you can do so; if not, the Sac. Lac. making no impression, the patients will inevitably resort to the *old treatment* before you have a chance to see what the arising symptoms are like. If the patients will not do this, send them away; they will soon come back to you; if not, the loss of their patronage will do less harm to your reputation as a good homœopath, than the *bad prescription* you would have been forced to make *without symptoms.*

Again, in cases of chronic constipation, you must require them to go without medication for a time, that you may find the especial symptoms to prescribe upon. You will find that they have habitually taken this, that, or the other compound, or an enema, every day, to produce action of the bowels; so they have but one symptom to tell you, and that is constipation. If you study the *Materia Medica* carefully, you will find that upon that symptom alone you could prescribe any or every drug that has ben proven, as all have it in greater or less degree. The patien t will insist that it will make him sick; but you may confidently assure him that it will *not*, for before he reaches that point you will have found the remedy. He wont die *until he is sick.* He wont be sick *until he has symptoms*, and when he has developed the latter you see the remedy, and before fifteen or eighteen hours are gone he will have had a natural motion of the bowels, and *without* suffering. Let him visit you every day for a week, study the case as it develops, there can hardly be a doubt that in the end you will have a perfect image of his sickness, and not only *get*, but *deserve*, the everlasting gratitude of your patient, and his whole family as your clients.

Again, a patient will come to you with prolapsus or one of the forms of version. She hears that you cure these conditions, and as her life is unhappy in the extreme, she hopes you will undertake her case. She is wearing a pessary. She will tell you she cannot walk a block without its support; that she cannot go up stairs; that she dare not take it out. Here you must be firm; you cannot prescribe for a pessary, nor can you deliberately feed her, one after another, such drugs as have especial relation to prolapsus or version. You must know how, when, and where she suffers, from the crown of her head to the sole of her foot, and you must find a drug to fit *that* condition. You do not want the language of pathology or of the doctor to tell you this. You want the simple, grand language of *nature* and of *the woman.* The men who prescribe upon any other basis don't think, don't reason, and won't wait to let symptoms so essential to a good prescription appear. You are interested in the pathological conditions, and it is quite right you should be, for Hahnemann in Section 3 of the *Organon* insists that the physician shall so acquaint himself with diseases that he may know the condition which is curable from the condition which is incurable. Again, Sęction 5. That he shall learn by the history of the case the probable causes, removing such as are possible, and then be able to interpret the symptoms developed, which are the *only* indications of the present disease expression. But you must not confound your knowledge of probable lesions and of the diagnosis of disease with the prescription or diagnosis of remedy, for in Section 7, with the former admonitions well in mind, he again says: "The totality of symptoms must be the chief and only means by which the disease is to make known the remedy for its cure." Make your prescriptions first upon the totality of symptoms, *then* diagnose the disease.

You have a patient with disease of the thorax, the same rule holds good; make first the *diagnosis of the remedy*, then the physical examination and diagnosis of diseased conditions—what the patient has seen, what the patient has felt are the only guide. Should you make your physical examination before you have prescribed, you will surely be biased by the physical changes found to have taken place, and fail to hit the case. Again, the etiology and history of a case must not be confounded with the pathology or diseased conditions, which relate entirely to the varying tissue changes, extending from a slight congestion to utter destruction of tissue, as in necrosis, cancer, etc.

Pathology and pathological conditions have thus far been total *failures* upon which to base a prescription. It is the symptoms that are needed, the symptoms which tell you of the modalities of the present and predict the future pathological conditions. We have honest reports of many *bona fide* cures of cancer, but these cures have not been made upon the pathological conditions of the cancer. for when disease teaches such, a point of destruction becomes localized, the symptoms are often swallowed up in the present manifestation. Such cures as have been made have either been made through what is called the pre-historic symptoms (such as were present, but have disappeared with the localization), or through symptoms such as are yet present and have not been eradicated by the disease. So learn here and now to treat the *patient* rather than the measles, and *not* the measles because it has got the patient.

These finer details and individualities, these shades of variation and modalities are the only bases upon which to obtain the requisite knowledge of drugs or of good prescribing. We must know the remedies as the woodsman knows his game. He knows by the simplest of signs that game is present, has been present within a short time; what kind, what habit; even the reason of its presence or its motions; he knows its haunts, its foot-prints, its character, whether bold or shy, clever, insiduous, or stupid; and from that knowledge of looks and habits can pretty accurately prognosticate its future movements. What to your untrained eye is but the flutter of dried leaves or dust from the breeze, to his accustomed eye is the flutter of partridge-wing or the alluring rustle of some mother-bird, whose nest you have approached too nearly. So in disease, we must know its objective and subjective symptoms, its method and manner of progression; then we must know the drugs that will produce such symptoms, conditions, and modalities. Symptoms that to the unaccustomed eye seem but shadows, as whether the patient is warm or cold; is better by application of cold or heat; what *agg.* the pains of this drug, what *amel.* them in another. You would hardly give a patient who was cold and anemic such remedies as belong to a class that are always too warm, want doors and windows open, and have high degree of arterial excitation, if every symptom that had been developed was covered by the remedy. We can hardly *know* and become familiar with remedies in both their noxious and their curative power without a strong *personal* interest. My advice is, cultivate that interest.

S. L. G. L.

